# Adoptive Infusion of Tolerogenic Dendritic Cells Prolongs the Survival of Pancreatic Islet Allografts: A Systematic Review of 13 Mouse and Rat Studies

**DOI:** 10.1371/journal.pone.0052096

**Published:** 2012-12-18

**Authors:** Guixiang Sun, Juan Shan, Youping Li, Yanni Zhou, Yingjia Guo, Wenqiao Wu, Tong Yang, Mengjuan Xia, Li Feng

**Affiliations:** 1 Key Laboratory of Transplant Engineering and Immunology of Health Ministry of China, West China Hospital, Sichuan University, Chengdu, Sichuan Province, People's Republic of China; 2 Regenerative Medical Research Center, West China Hospital, Sichuan University, Chengdu, Sichuan Province, People's Republic of China; 3 Chinese Cochrane Centre, Chinese Evidence-Based Medicine Centre, West China Hospital, Sichuan University, Chengdu, Sichuan Province, People's Republic of China; University of Michigan Medical School, United States of America

## Abstract

**Objective:**

The first Phase I study of autologous tolerogenic dendritic cells (Tol-DCs) in Type 1 diabetes (T1D) patients was recently completed. Pancreatic islet transplantation is an effective therapy for T1D, and infusion of Tol-DCs can control diabetes development while promoting graft survival. In this study, we aim to systematically review islet allograft survival following infusion of Tol-DCs induced by different methods, to better understand the mechanisms that mediate this process.

**Methods:**

We searched PubMed and Embase (from inception to February 29^th^, 2012) for relevant publications. Data were extracted and quality was assessed by two independent reviewers. We semiquantitatively analyzed the effects of Tol-DCs on islet allograft survival using mixed leukocyte reaction, Th1/Th2 differentiation, Treg induction, and cytotoxic T lymphocyte activity as mechanisms related-outcomes. We discussed the results with respect to possible mechanisms that promote survival.

**Results:**

Thirteen articles were included. The effects of Tol-DCs induced by five methods on allograft survival were different. Survival by each method was prolonged as follows: allopeptide-pulsed Tol-DCs (42.14±44 days), drug intervention (39 days), mesenchymal stem cell induction (23 days), genetic modification (8.99±4.75 days), and other derivation (2.61±6.98 days). The results indicate that Tol-DC dose and injection influenced graft survival. Single-dose injections of 10^4^ Tol-DCs were the most effective for allograft survival, and multiple injections were not superior. Tol-DCs were also synergistic with immunosuppressive drugs or costimulation inhibitors. Possible mechanisms include donor specific T cell hyporesponsiveness, Th2 differentiation, Treg induction, cytotoxicity against allograft reduction, and chimerism induction.

**Conclusions:**

Tol-DCs induced by five methods prolong MHC mismatched islet allograft survival to different degrees, but allopeptide-pulsed host DCs perform the best. Immunosuppressive or costimulatory blockade are synergistic with Tol-DC on graft survival. Multiple injections are not superior to single injection. Yet more rigorously designed studies with larger sample sizes are still needed in future.

## Introduction

Transplantation is the most effective therapy for end-stage organ failure. However, clinical success of organ transplantation has been achieved through nonspecific immunosuppressive drugs that inhibit the immune response [1]. These drugs have many side effects that can increase the risk of cardiovascular disease, infection, and cancer. Thus, there is an urgent need to find new ways to induce organ-specific immune tolerance without affecting recipients' normal immune defense.

In recent years, more and more studies have focused on the prospective value of dendritic cells (DCs) to induce clinical organ transplant tolerance, as well as prolong graft survival [2], [3], [4]. DCs are rare, uniquely well-equipped, functionally diverse professional antigen-presenting cells (APCs) [3]. They play a key role in innate and adaptive immunity, and are essential for tolerance induction. DC function is closely related to their stage of differentiation, activation, and maturation [4]. During organ transplantation, tolerogenic DCs (Tol-DCs) favor graft acceptance. They are characterized by increased expression of CCR7, CCR5, CCR6, and other chemokine receptors, decreased expression of major histocompatibility complex II (MHC-II), and costimulatory molecules such as CD80 and CD86 [31]. Tol-DCs induce T cells hypo-reactivity, drive the generation of T regulatory cells (Treg), and induce antigen-specific immune tolerance.

Type I diabetes (T1D) is an autoimmune disease characterized by T cell-mediated destruction of insulin-producing beta cells [5]. Yet, the first phase I (safety) study of autologous Tol-DCs in T1D patients was recently published [6]. Tol-DCs also protect allografts, which highlight their application for pancreatic islet transplantation in the clinic. We therefore conducted a systematic review of pancreatic islet allograft survival affected by Tol-DC adoptive infusion to provide new ideas for long-term graft survival, better understand the mechanisms involved, and to advance their clinical application.

## Materials and Methods

### Publication search and inclusion criteria

PubMed and Embase (from inception to February 29^th^, 2012) were searched for relevant studies with the following MeSH headings or text words: “dendritic cells”, “pancreas islet transplantation”.

Studies meeting the following criteria were included: (1) Chinese or English publication, (2) pancreatic islet transplant recipients as the target population, and (3) the study objective was to evaluate the effect of Tol-DC adoptive infusion on graft survival. Review articles, abstracts, and *in vitro* studies were excluded. If the same group published similar data, we included only the study containing the most complete information.

### Quality assessment

We rated study quality on six criteria as follows [7], [8]: (1) peer reviewed publication (2 scores), (2) random allocation to treatment or control (2 scores), (3) animal species (inbred strain, age-matched, statement of MHC mismatch, 2 scores), (4) sample size (sample size of both control and experimental groups must be clearly defined, 1 score), (5) animal welfare regulations were observed (1 score), and (6) statement of potential conflict of interests (funding sources must be clearly stated, 1 score). If information was incomplete in any criteria, the score was assigned half of the corresponding score. Study quality was stratified into four ranks according to their scores, which ranged from 0 to 9: ≥7 was ranked as A, <7 and ≥5 as B, <5 and ≥3 as C, <3 as D. If a study was conducted using inbred animal models, we considered it equivalent to a random allocation in the absence of individual heterogeneity. Discrepancies were resolved by Y.L or J.S.

### Data extraction

Two reviewers independently extracted data from the selected articles. We extracted data on animal model, methods of inducing Tol-DCs, source of Tol-DCs, time, route of administration, frequency and dose of Tol-DC administration, allograft survival and the potential mechanisms of interest. Important unpublished data were obtained by contacting corresponding authors whenever Possible.

Discrepancies between these two reviewers were resolved by the third reviewer.

### Data analysis

Allogeneic pancreatic islet graft survival time was used to assess endpoint outcomes. Meta-analysis could not be used because of incomplete data in most studies. We displayed survival time of both experimental and control groups as 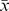
±SD in a forest map, as described previously [9]. Immune tolerance was defined when survival time exceeded 100 days, based on induction of donor specific T cell hyporesponsiveness (MLR), skewing of Th0 to Th2 (CK), induction of CD4^+^CD25^+^ regulatory T cells (Treg), and reduction of cytotoxicity against allografts (CTL). We dissected the effects of Tol-DC adoptive transfusion on islet allografts and evaluated potential survival mechanisms.

## Results

### Literature search and selection

**Figure 1:**
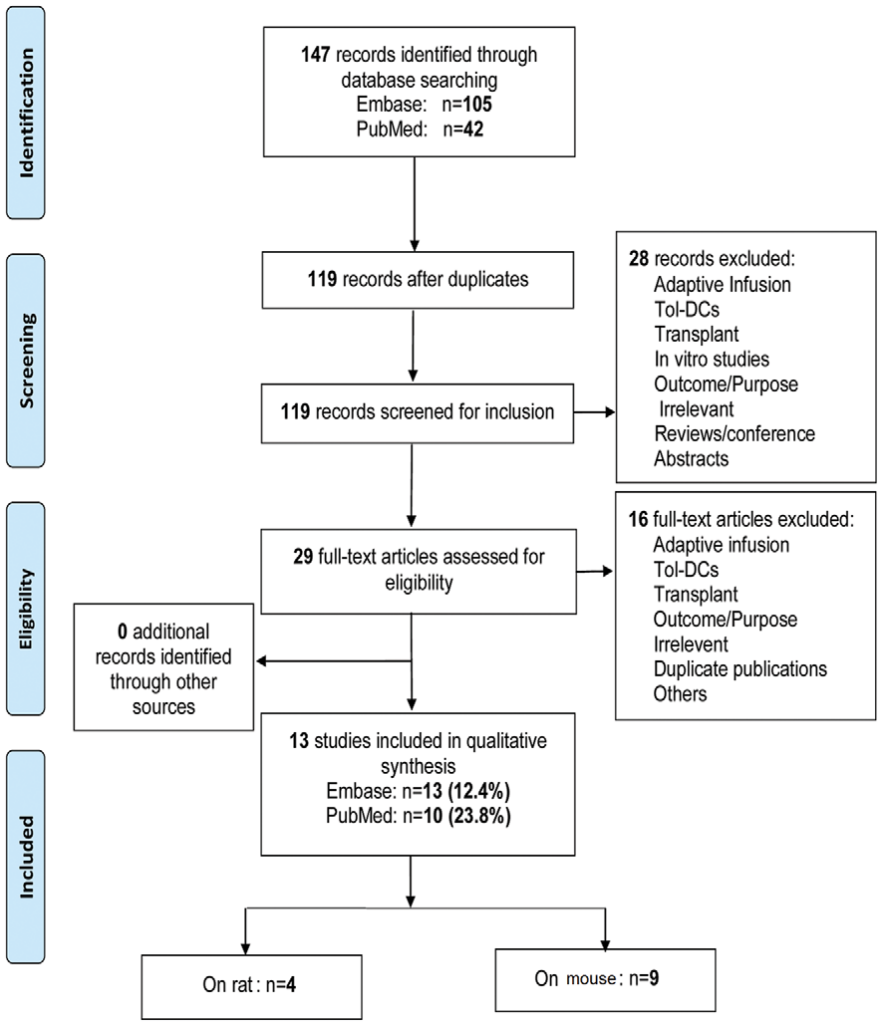
Flow diagram for selection of studies.

147 relevant studies were identified, consisting of 105 from Embase and 42 from PubMed. To our knowledge, there has not been a systematic review of the literature using similar criteria. We selected 13 studies according to the above inclusion criteria, which included adoptive mouse (9 articles) and rat (4 articles) islet transplantation models [10], [13], [14], [15], [16], [19], [20], [21], [22], [11], [12], [17], [18]. The detection rate in PubMed and Embase was 23.8% (10 articles) and 12.4% (13 articles), respectively (Figure 1).

### Quality of included studies

The 13 studies included scores ranging from 4 to 9, and contained 11 studies ranked A [10], [11], [12], [13], [14], [15], [18], [19], [20], [22], one ranked B [17], one ranked C [21] and none ranked D (Table 1). Generally, the quality of included studies was high in these criteria.

**Table 1:** Quality assessment of included studies.

No.	Study	1	2	3	4	5	6	Score	Grade
		(2 scores)	(2 scores)	(2 scores)	(1 score)	(1 score)	(1 score)		
1	Stepkowski(2006)1	√	√	√	√	-	√	8	A
2	huang(2010)7	√	√	√	-	√	√	8	A
3	Hauben(2008)6	√	√	√	√	-	√	8	A
4	Olakunle(2001)11	√	√	√	√	-	√	8	A
5	Ali(2000)12	√	√	√-	√	-	√	7	A
6	Oluwole(1995)13	√	√	√-	√	-	√	7	A
7	Yang(2008)2	√	√	√	√	-	√	8	A
8	Zhu(2008)3	√	√	√	√	-	√	8	A
9	O'Rourke(2000)4	√	√	√	√	√	√	9	A
10	Li(2010)5	√	-	√-	-	√	-	4	C
11	Kim(2006)8	√	√	√	-	-	√	7	A
12	Rastellini(1995)9	√	√	√	√	-	√	8	A
13	Chaib(1994)10	√	√	√-	√	-	-	6	B

“-”Articles did not report relevant information. “√-”Articles reported partial information.

Criteria: (1) Peer reviewed publication. (2) Random allocation of treatment and control. (3) Animal species (inbred line, age-matched, MHC mismatch). (4) Sample size calculation (sample size of both control and experimental groups must be clarified). (5) Compliance with animal welfare regulation. (6) Statement of potential conflict of interest (source of funds must be clarified).

### Characteristics of included studies

#### Interventions

Six methods were reported to induce Tol-DCs. The most commonly used-method was gene modification (4 articles, accounting for 30.76%), followed by allopeptide-pulsed (3 articles, 23.07%), other derivation (3 articles, 23.07%), immature dendritic cells (imDC) (1 article, 7.69%), drug intervention (1 article, 7.69%), and mesenchymal stem cell (MSC) induction (1 article, 7.69%) (Table 2).

**Table 2:** Characteristics of included studies.

NO.	Study	Animal model	(Mice/Rat)		Tol-DC(Number)(total number)	Controls		Outcomes					DC(R/D)
						C1	C2	O1	O2	O3	O4	O5	
						Untreated	Negative	SUR	MLR	CK	Treg	CTL	
A1	* Stepkowsk(2006)1	(D)H-2^b^	(R)H-2^d^	(T)H-2^k^	Bioreactor-imDC(Balb/c) (5)	√		>150d	/	/	Y	/	R-DC
					Bioreactor-imDC(Balb/cStat4−/−) (5)			>150d					
	Totle	MHC total mismatch: n = 1			Monotherapy: n = 0	1	0	1	0	0	1	0	R-DC:n = 1
					Combination: n = 1								D-DC:n = 0
B1	Olakunle(2001)11	(D)RT-1^u^	(R)RT-1^a^	(T)RT-1^n^	P5-BMDC(10∧6,i.v.) (5)	√		↑	Y	/	/	/	R-DC
					P5-BMDC+ALS(2*10∧6,i.v.) (5)			>200d					
					P5-BMDC(2*10∧6,i.v.) (4)			↑					
					P5-BMDC+ALS(10∧6,i.t.) (11)			>200d					
					P5-thymic DC(5*10∧6,i.v.) (4)			↑					
					P5-thymic DC+ALS(5*10∧6,i.v.) (4)			>200d					
B2	Ali(2000)12	(D)RT-1^u^	(R)RT-1^a^	(T)RT-1^n^	P5-DC+ALS(-) (5)	√	√	↑	Y	/	/	/	R-DC
					P5-DC+ALS(0.5 ml) (5)			↑					
B3	Oluwole(1995)13	(R)RT-1^u^	(D)RT-1^l^	(T)RT-1^n^	D-Ag+DC(R) (3)	√	√	↑	Y	/	/	/	R/D-DC
					D-Ag+DC(D) (4)			-					
	Totle	MHC total mismatch: n = 3			Monotherapy: n = 3	3	2	3	3	0	0	0	R-DC:n = 3
					Combination: n = 2								D-DC:n = 1
C1	Yang(2008)2	(R)H-2^b^	(D)H-2^d^		CTLA-4Ig-DC(8)	√	√	↑	Y	TH2	/	/	/
C2	Zhu(2008)3	(R)H-2^b^	(D)H-2^d^		IL10-DC(8)	√	√	↑	Y	TH2	/	/	R-DC
C3	O'Rourke(2000)4	(R)H-2^b^	(D)H-2^d^	(T)H-2^k^	D2SC/1-CTLA4-Ig (10)	√	√	↑	Y	/	/	/	D-DC
					D2SC/1-CTLA4-Ig (additional injection)			-					
C4	& Li(2010)5	/	/		rAd-DCR3-DC	√	√	↑	/	/	/	Y	/
					rAd-GAD65/DCR3-DC			↑					
	Totle	MHC total mismatch: n = 3			Monotherapy: n = 4	4	4	4	3	2	0	1	R-DC:n = 1
					Combination: n = 0								D-DC:n = 1
D1	Hauben(2008)6	(D)H-2^b^	(R)H-2^d^		mDC-VAF347 (17)	√	√	↑	Y	TH2	Y	/	R-DC
					imDC+VAF347 (19)			-					
					mDC (14)			-					
					imDC (18)			-					
	Totle	MHC total mismatch: n = 1			Monotherapy: n = 1	1	1	1	1	1	1	0	R-DC:n = 1
					Combination: n = 0								D-DC:n = 0
E1	& Huang(2010)7	(R)H-2^b^	(D)H-2^d^		R-KSC+D-DC	√	√	↑	Y	-	-	/	R/D-DC
					R-KSC+R-DC			-					
	Totle	MHC total mismatch: n = 1			Monotherapy: n = 1	1	1	1	1	1	1	0	R-DC:n = 1
					Combination: n = 0								D-DC:n = 1
F1	Kim(2006)8	(R)H-2^b^	(D)H-2^d^	(T)H-2^k^	CD4^+^imDC+anti-CD154Ab (6)	√	√	>120d	Y	TH2	Y	/	D-spleen DC
					CD4^+^imDC+antiCD154Ab+anti-IL10R Ab(4)			>120d					
					CD4^+^imDC (6)			-					
					CD8^+^imDC (6)			-					
					CD8^+^imDC+anti-CD154Ab (6)			-					
F2	Rastellini(1995)9	(R)H-2^b^		(D)H-2^k^	liver-imDC(10)	√	√	↑	Y	/	/	/	D-liver DC
					spleen-imDC (4)			-					
F3	Chaib(1994)10	(D)RT-1^u^	(R)RT-1^l^		DC+ALS (9)	√		-	/	/	/	/	D-spleenDC
					NPC+ALS (8)			-					
	Totle	MHC total mismatch: n = 3			Monotherapy: n = 3	3	2	3	2	1	1	0	R-DC:n = 0
					Combination: n = 1								D-DC:n = 3

A1: Immature dendritic cells (imDC) group. B1–3: Allopeptide-pulsed group. C1–4: Gene modification group. D1: Drug intervention group. E1: Mesenchymal stem cell (MSC) induction group. F1–3: Other derived group.

“&” Articles did not report the sample size. “/” Articles did not report relevant information. “-” No difference between experiment group and control group.

H-2^b^: C57. H-2^d^: BAL/C. H-2^k^: C3H. RT-1^u^: WF/WAG. RT-1^a^: ACI. RT-1^n^: BN. RT-1^l^: Lewis.

D: Donor. R: Recipient. T: The third party. MHC: Major histocompatibility complex. BMDC: Bone marrow dendritic cell. Ag: Antigen. R-KSC: Host kidney-derived MSC. NPCs: Non-parenchymal cells. ALS: Anti-lymphocyte serum. P5: MHC Class I peptide five. D-DC: Donor-derived DC. R-DC: Recipient-derived DC. SUR: Survival, “↑” Prolongation. MLR: Mixed lymphocyte reaction, “Y” Successfully induced donor specific T cell hyporesponsiveness. CK: Cytokine. CTL: Cytotoxic T lymphocyte, “Y” Reduced cytotoxicity against allografts. Treg: Regulatory T cells, “Y” Successfully induced Treg.

#### Animal model

Eight studies adopted MHC mismatched inbred mice models, with four MHC mismatched inbred rat models (Table 2).

#### Experimental design

Eight articles studied Tol-DCs monotherapy, and 4 articles studied the synergistic effect of immunosuppressive agents or costimulatory blockade with Tol-DC. Seven articles used recipient-derived DCs, six used donor-derived DCs, and another two did not report the DC source. Routes of administration were intravenous (i.v., six articles), intrathymic (i.t., three articles), intraperitoneal (i.p., two articles), subcutaneous (s.c., one article). The Tol-DC doses administered varied form from 10^4^ to 10^7^ cells. Nine studies adopted single-injection, and three used multiple injections. All untreated groups were taken as control groups, and only ten studies had negative control groups (Table 2).

#### Outcomes

Prolonged graft survival was reported in 11 of 13 studies, and two reported rejection episodes. Similarly, 10 studies detected Tol-DC induced donor-specific T cell hyporesponsiveness against donor antigens by MLR, 6 detected Th1/Th2 differentiation, 4 detected Treg induction, but only one detected anti-graft cytotoxicity (Table 2).

### Outcomes

#### imDC prolonged allografts survival

**Figure 2:**
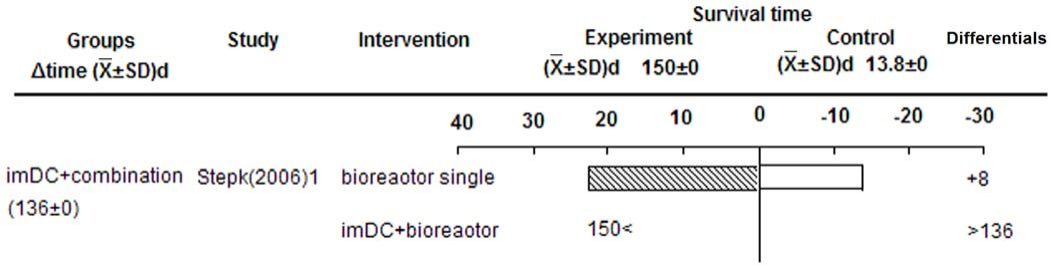
Effects of imDC on islet allograft survival. Key information is displayed for each group as follows (L to R): Group name and average survival extension (days) in each group, included study name, main intervention method, survival time of experimental and control groups, survival differentials between experimental and control groups, and P-value from original study (where possible). This structure in describing the figure also applies to the following figures.

imDC prolonged islet allograft survival when incubated in a special bioreactor with continuous rotation in culture media, and even appeared to induce immune tolerance (>100 d) (Figure 2). We speculate that Tol-DCs increased generation of donor-specific CD4^−^CD25^−^ Treg cells in recipients transplanted with allogeneic islets depleted of donor “passenger” DCs, after cultured in bioreactors [10].

#### Allopeptide-pulsed host Tol-DCs prolonged graft survival

**Figure 3:**
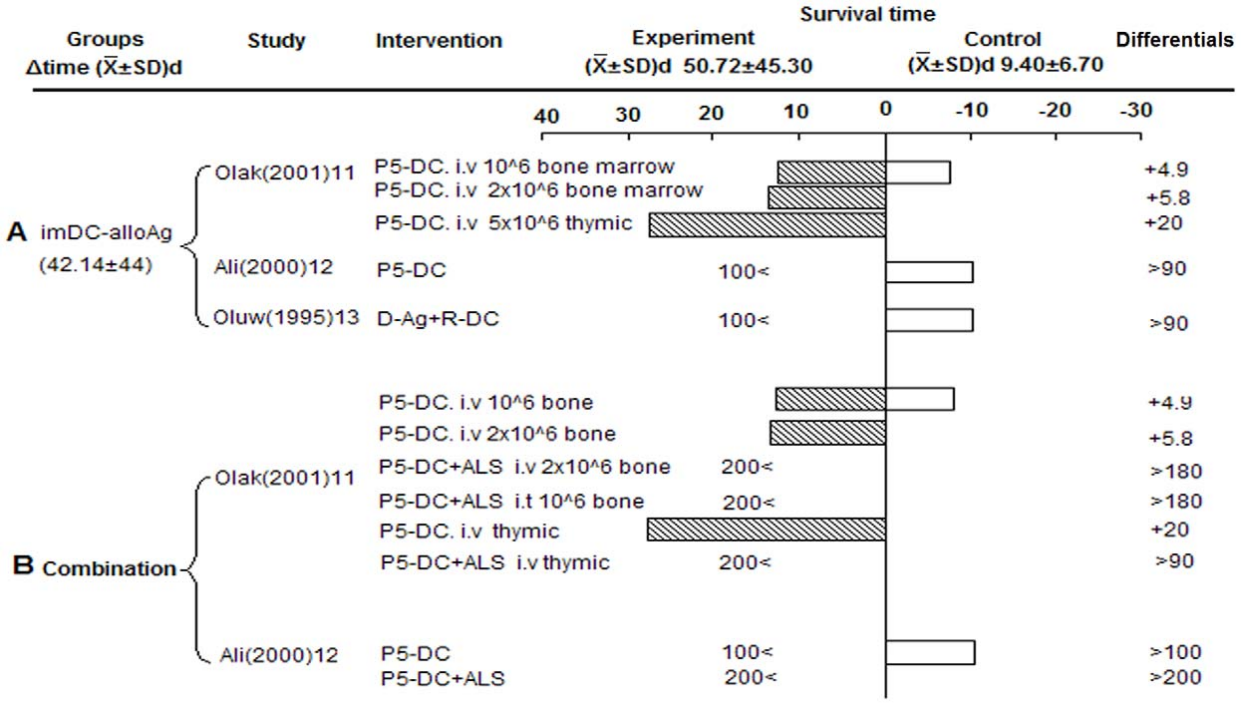
Effects of allopeptide-pulsed host Tol-DCs on islet graft survival. A) Single-injection of alloAg-imDC. B) AlloAg-imDC plus ALS group. imDC-alloAg: Allopeptide-pulsed imDC.

Three studies adopted a rat islet transplantation model with an intrathymic route of administration. Infusion allopeptide-pulsed host Tol-DCs prolonged graft survival compared to controls (42.14±44 d) (Figure 3 A). However, we could not rule out positive effects of intrathymic injection on graft survival. Interestingly, Oluwole et al reported donor-derived DCs did not favor survival, which was opposite to results observed with recipient-derived DCs [11]. One study reported the synergistic effect of anti-lymphocyte (ALS) serum with Tol-DCs, which showed marked prolongation of permanent islet allograft survival (>100 days) (Figure 3 B) [12].

#### Drug intervention of Tol-DCs prolonged graft survival

**Figure 4:**
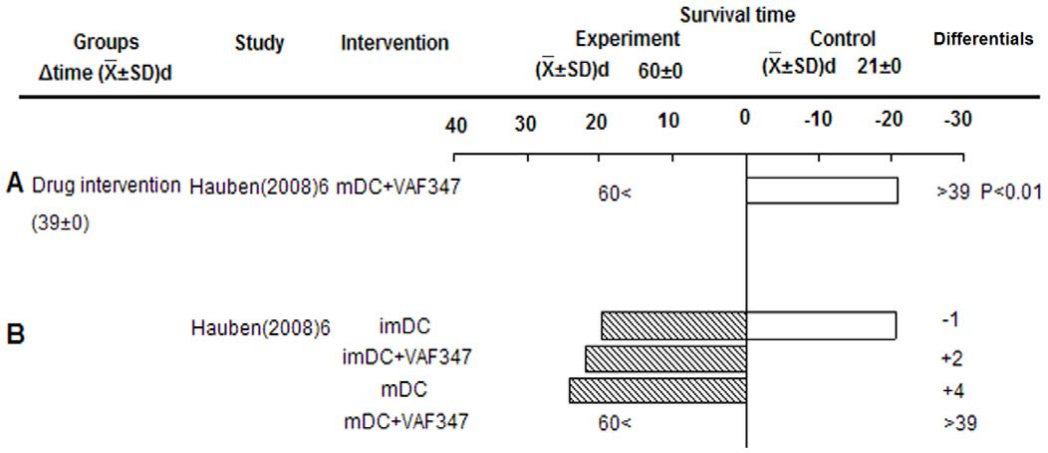
Effects of drug intervention Tol-DCs on islet allograft survival. A) mDC+VAF347. B) imDC or mDC with/without VAF347. VAF347: VAF347 is a low-molecular-weight compound, which can modify immune responses through induction of Tol-DC, and activating AhR resulted in induction of Foxp3^+^Treg cells.

As shown in Figure 4, drug treatment of Tol-DCs significantly prolonged the average survival to over 39 days compared to the control group (P<0.01, derived from original study). They demonstrated that infusion of mature dendritic cells (mDC) and imDC without drug treatment showed no obvious effect on islet allograft survival, and mature, but not immature, VAF347-BMDCs could promote long-term islet allograft survival (Figure 4 B). The authors speculated that VAF347-treated imDCs were ineffective because they encountered *in vivo* pro-inflammatory signals, which reversed their tolerogenic phenotype [13].

#### Mesenchymal Stem Cell (MSC) induction of Tol-DCs prolonged graft survival

**Figure 5:**
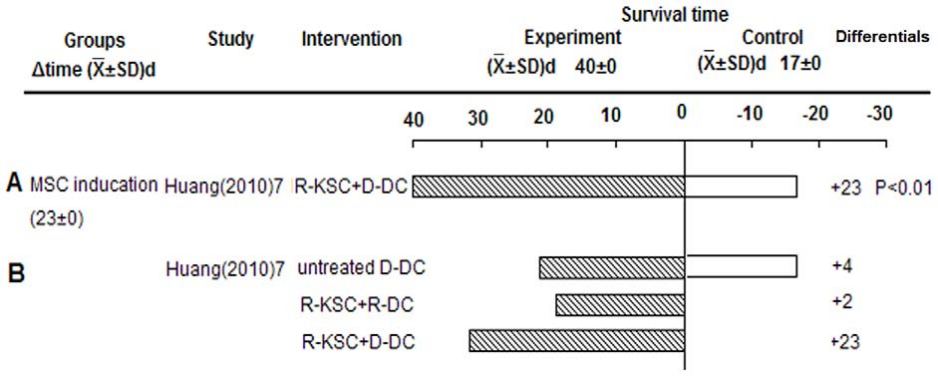
Effects of MSC-induced Tol-DCs on islet allograft survival. A) Recipient-KSC+Donor-DC. B) Recipient-KSC+Recipient/Donor-DC.

Donor but not recipient DCs, cultured with host kidney-derived MSCs (KSCs), prolonged islet allograft survival (23 days, P<0.01, derived from original study) (Figure 5 A, B). The effect of intervening with DCs between donor and recipient on graft survival was different from that observed by Oluwole et al with the allopeptide-pulsed group. This suggests that the mechanism of each intervention method may be worth investigating. Coculture largely induced a DC phenotype (KSC-DC) with reduced MHC-II expression, increased CD80 expression, and the ability to suppress T cell responses [14]. Co-cultured recipient DCs failed to promote graft survival, which may be related to the strength of the direct allorecognition pathway being activated early after transplantation.

#### Gene modification of Tol-DCs prolonged graft survival

**Figure 6:**
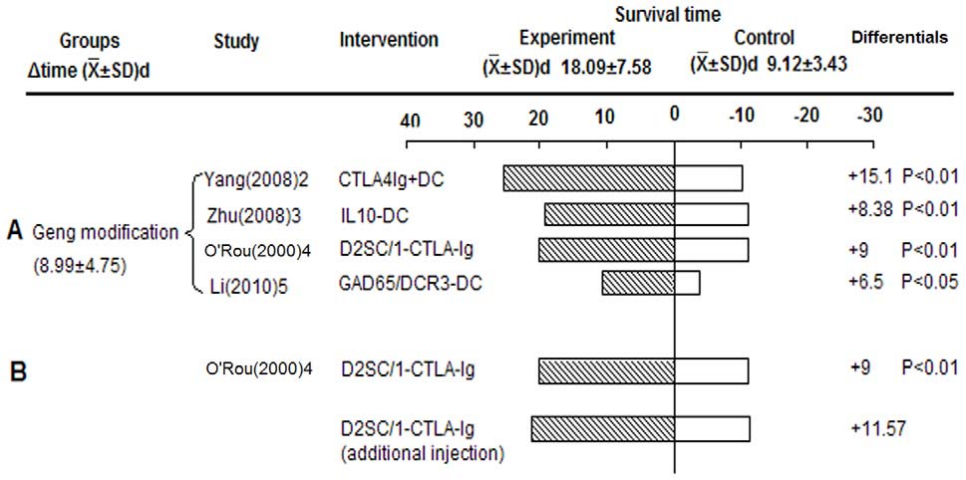
Effects of gene-modified Tol-DCs on islet allograft survival. A) Survival of gene modification group. B) Effect of additional injections on allograft survival. D2SC/1: A transformed murine dendritic cell line of BALB/c origin, which presents class II-restricted antigens *in vitro* and class I antigens *in vitro* and *in vivo*.

Different gene-modified Tol-DCs such as those targeted on CTLA-4, IL-10, and GAD65/DCR3, significantly prolonged survival compared to controls (8.99±4.75 d, P<0.05, derived from original study) (Figure 6 A). Unexpectedly, O'Rourke et al demonstrated that the addition of three preoperative doses of cells to the two peri-operative ones did not result in a significant increase in allograft survival, compared with the regimen consisting of only two peri-operative doses [15] (Figure 6 B). This suggests that additional injections did not contribute more to promoting survival, but instead increased the risk and cost.

#### Other derived Tol-DC prolonged grafts survival

**Figure 7:**
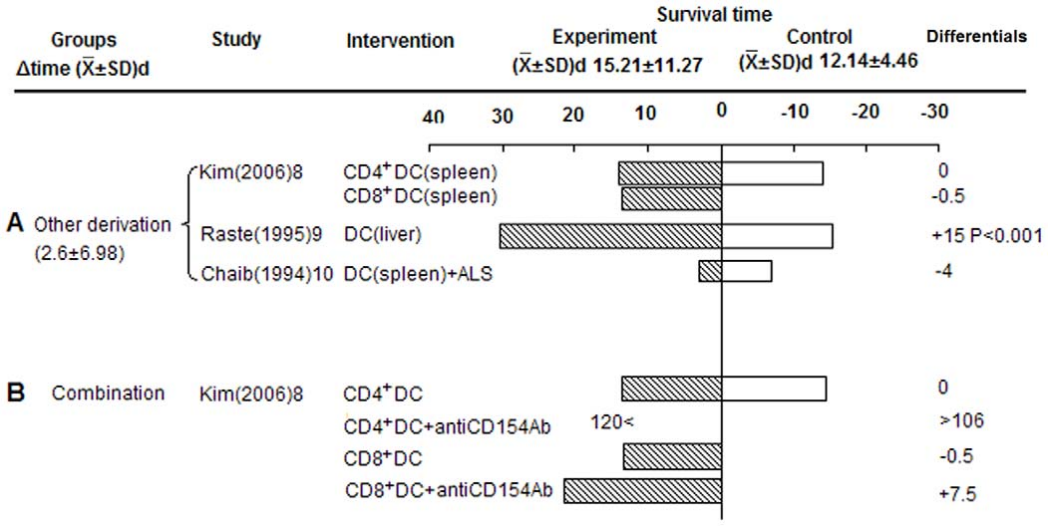
Effects of other derived Tol-DCs on islet allograft survival. A) Survival of other derived group. B) Survival of combination therapy group (CD4^+^DCs or CD8^+^DCs plus anti-CD154Ab).

Three studies used Tol-DCs derived from donor spleen or liver. Compared to controls, Tol-DCs prolonged graft survival (2.6±6.89 d), while only liver-derived DCs favored islet allograft survival, and donor spleen-derived DCs showed rejection episodes in two studies (Figure 7 A). Kim et al. demonstrated pre-treatment of hosts with either CD4^+^DCs or CD8^+^DCs did not produce prolonged islet allograft survival compared with controls, but did prolong survival when combined with antiCD154Ab [16] (Figure 7 B). Furthermore, the provision of anti-CD154Ab plus CD4^+^DCs created tolerance, but not CD8^+^DCs (Figure 7 B). This suggests that DC subsets and co-stimulatory signals play an important role on graft survival. Beyond that, Chaib et al. reported animals receiving intrathymic inoculation with liver non-parenchymal cells (NPC) or spleen DCs plus ALS, rejected islet allografts. This is in contrast to their previously published work where tolerance to cardiac grafts was induced by intrathymic NPC inoculation under transient immunosuppression with ALS, prompting them to speculate that organ-specific tolerance was induced by NPC through their experimental protocol [17].

#### Summary of main results

According to GRADE standards, we made a summary table of the key results to summarize the data and effect sizes of important endpoint outcomes (Table 3). Although gene modification of DCs was the most commonly used-method in MHC mismatched islet allografts, allopeptide-pulsed Tol-DCs were most effective at prolonging survival. The main route of administration in this model was intravenous, but intrathymic injection showed the best results. Single-injection dose of 1–9×10^5^ DCs was most commonly used, yet the10^4^ group clearly prolonged survival compared with other doses. Multiple injections did not make substantive contributions to promoting survival, but instead increased the risks and costs. Due to the limited number of studies included and incomplete data, more high quality studies with larger sample sizes are still needed.

**Table 3:** Summary table of findings.

				Summary of findings		
				Differentia	Absoute effect	
Category	Outcome concerned (n)	Quality assessment	Total number (n)	( 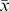 ±SD)d	Tol-DC( 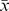 ±SD)d	Control( 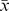 ±SD)d
**Different methods**	imDC(1)	A(1A)	15	128.40±0.00	150.00±0.00	13.80±2.70
	imDC-alloAg(3)	A(3A)	87	42.14±44.00	50.72±45.30	9.40±6.70
	Drug intervention(1)	A(1A)	80	39.00±0.00	60.00±0.00	21.00±0.00
	MSC inducation(1)	A(1A)	24–48*	23.00±0.00	40.00±0.00	17.00±0.00
	Gene modification(4)	A(3A1C)	80	8.99±4.75	18.09±7.58	9.12±3.43
	Other derivation(3)	A(2A1B)	98	2.61±6.98	15.21±11.27	12.14±4.46
**Injection pathways**	i.t.(3)	B(2A1B)	66	58.62±60.81	67.66±56.00	9.21±1.93
	i.v.(6)	A(6A)	203	15.87±14.09	29.63±18.04	13.77±4.71
	i.p.(2)	A(2A)	61	11.73±4.75	22.44±4.30	10.72±0.42
	s.c.(1)	C(1C)	-*	6.50±0.00	10.50±0.00	4.00±0.00
**Does of DC**	(S)10^4^(1)	A(1A)	21	90.00±0.00	100.00±0.00	10.30±1.10
	(S)1–9×10^5^(4)	A(3A1B)	158	31.17±43.57	44.25±44.62	13.07±6.00
	(S)1–9×10^6^(3)	A(3A)	95–119*	15.73±8.90	27.93±11.67	12.20±4.66
	(S)10^7^(1)	A(1A)	33	15.10±0.00	25.52±17.99	10.42±2.76
	(M)5×10^5^(1)	C(1C)	-*	6.50±0.00	10.50±0.00	4.00±0.00
	(M)10^7^(1)	A(1A)	28	8.38±0.00	19.38±9.81	11.00±1.58
	(M)2×10^7^(1)	A(1A)	19	9.00±0.00	20.00±9.39	11.00±1.14
**Animal model**	Rat(4)	A(3A1B)	110	45.30±51.42	54.12±53.14	8.82±1.73
	Mouse(8)	A(7A1C)	244–268*	14.55±12.46	27.08±16.59	12.54±5.02
**Source of DC**	Donor-Ag pulsed R-DC(3)	A(3A)	87	61.73±48.44	71.16±49.94	9.43±1.50
	Recipient-derived DC(3)	A(3A)	132–156*	23.46±15.32	39.79±20.31	16.33±5.03
	Donor-derived DC(5)	A(4A1B)	126–150*	9.28±11.55	21.46±14.32	12.18±3.70
	Donor-Ag pulsed D-DC(1)	A(1A)	21	−4.00±0.00	6.30±0.00	10.30±0.00

S: Single injection. M: Multiple injections.

“*” Sample size of one study is a range, not exact number. “-*” Article did not report sample size.

#### Possible mechanisms underlying Tol-DCs prolonging graft survival

Infusion of Tol-DCs likely prolonged islet allograft survival through the following five mechanisms (Table 2): (1) Induction of T cells donor-specific hyporesponsiveness via T cell deletion and/or anergy. Of the ten studies reporting MLR, nine showed positive results and prolonged islet graft survival. (2) Skewing of Th0 to Th2. Of the six studies reporting a Th0 shift, five reported shifting to Th2, which appears to have prolonged survival. (3) Treg expansion. Two of the three Treg articles reported an increase in Treg. (4) Decreased cytotoxicity against grafts. One study showed positive results with respect to prolonged survival. (5) Induced chimerism. One study reported potentially tolerogenic precursors of chimeric cells in islet allograft recipients, which might contribute to prolonged graft survival.

## Discussion

### Tol-DCs significantly prolong islet allograft survival through various mechanisms

Induction of maturation-resistant and stable tolerogenic DCs is a prerequisite for its application in the clinic. Our systematic review identified five kinds of Tol-DCs that had different effects on islet graft survival (Table 3). The route of injection also effected islet graft survival. Tol-DCs can promote allograft survival through both central and peripheral tolerance. Central tolerance is achieved through negative selection of self- or foreign Ag-reactive thymocytes, and is a highly efficient process mediated by APCs, which induce specific T cell anergy and Treg generation in the thymus [3]. Intrathymic injection of allopeptide-pulsed host DCs was the most effective way to promote graft survival (Table 3). This finding provided evidence for a direct link between indirect allorecognition in the thymus and the induction of acquired thymic tolerance. In this islet transplantation model, Tol-DCs maintained peripheral tolerance to self-Ags through various inter-related mechanisms. These mechanisms include inducing donor-specific T-cell hyporesponsiveness, production of immunoregulation factors such as IL-2, IL-4, INF-r and IL-10, skewing of Th0 to Th2, increasing Treg, decreasing anti-graft cytotoxicity, generation of chimerism, and inhibiting B cell responses and anti-donor Ab responses [12], [13], [16], [19], [20], [21]].

Tol-DCs are expected to not only prolong survival, but also induce tolerance. In all, five studies successfully induced tolerance. Three reported transfusions of allopeptide-pulsed host DCs via intrathymic injection that could maintain stable central tolerance [11], [12], [18]. Another study reported infusion of imDC plus islet graft cultured in a special bioreactor could induce immune tolerance [10]. Kim et al. found donor spleen-derived CD4^+^ DC plus anti-CD154Ab, which decreased IL12 expression, Th2 differentiation, Treg proliferation and induction, and was an effective way to induce tolerance [16]. Interestingly, KSC-DCs were characterized by reduced MHC-II but increased CD80 expression, and the ability to suppress T cell responses suggested a new stage for tolerance induction [14].

### Other critical factors related to islet allografts survival

In addition to different means of inducing Tol-DCs in allograft survival, there are several other critical factors related to survival:

1. **Prior to DC infusion.** The source of DCs (donor-derived, recipient-derived, semi-allogeneic), DC subsets (bone-marrow dendritic cells (BMDC), plasmacytoid DCs (pDC), CD4^+^DC, CD8^+^DC), recovery, and purity of the DC were important factors [3]. Actually, our outcomes suggested effect interventions with DCs between donor and recipient on graft survival were different, such as for the MSC induction and allopeptide-pulsed groups mentioned previously. BMDC and other derived DCs both prolonged islet graft survival (Table 3). However, it was different between liver-derived DCs and spleen-derived DCs. Rastellini et al. demonstrated liver-derived DCs, but not spleen-derived DCs, prolonged islet graft survival. This is likely because liver-derived DCs express lower levels of MHC-II and CTLA-4CR, which might account for their better effect on graft survival [22]. The inherent tolerogenicity of liver and chimerism were also critical reasons for graft survival [1].
2. **Tol-DCs infusion**. The time and route of administration, frequency and dose of Tol-DCs administered, and optimum combined immunosuppression (biological agents, pharmacological agents) should all be considered when evaluating results [3].
3. **Organ specificity.** As described above, Chaib et al. Demonstrated that non-parenchymal cells (NPCs) induced organ-specific tolerance in their experiments. Generally, the different organs related to immunogenicity, histology and physiology showed diversity and complexity in the same immune intervention [17].
4. **Non-immunologic factors.** These non-immunologic factors play a key role in graft survival, separately or synergistically, such as seen in ischemia-reperfusion injury (IRI), infection, disease stage, graft preservation, and operation difficulty [23], [24].

### Tol-DC therapy in clinical islet transplantation

DC vaccines have been applied successfully in clinical cancer therapy [25], [28], which highlights the feasibility of the clinical translation of Tol-DC in transplantation. Cell therapy with Tol-DC is already underway in human autoimmune disease [26]. The first Phase I (safety) study of autologous Tol-DCs in T1D patients was published recently [6]. The results show that DCs were tolerated, discernible adverse events did not occur in patients, and DCs up-regulated the frequency of B220^+^CD11c-B cells [6]. However, there are no reports regarding Tol-DC therapy in clinical islet transplantation. Although it has proven effective in mice [27], small animals and humans are different. There is still much to learn about the optimization of Tol-DC therapy for clinical islet transplantation, such as what dose, frequency, and route of administration to use, and the length of time appropriate for treating with Tol-DC. Even so, small animal models provide important insights into the mechanisms underlying tolerance induction [1], [29], [30]. We believe that Tol-DCs will one-day play a critical role in the treatment of clinical islet transplantation for T1D.

### Limitations of our review

Research on adoptive infusion of Tol-DCs prolonging islet graft survival is at an early stage, with results available in only a few select studies (13 in our systematic review). Descriptive analysis was conducted in this review, but not meta-analysis, due to incomplete data and little similarity between the studies selected. In addition, our results may have a bias due to small sample size and incomplete data in most studies. This systematic review only assessed the influence of adoptive transfusion of Tol-DCs on islet allograft survival. However, we have also conducted six systematic reviews on its effect in other organ transplantation models, which has been published [31] or are in preparation.

## Conclusions

In conclusion, Tol-DCs induction by different mechanisms prolonged MHC mismatched islet allograft survival to different degrees, but allopeptide-pulsed host DCs performed the best. Immunosuppressive or costimulatory blockade were synergistic with Tol-DC on graft survival, and could even help induce immune tolerance. A single-intrathymic injection of 10^4^ Tol-DCs prolonged survival more than other doses. Multiple injections were not more effective at promoting survival yet increased the risk and cost.

## Supporting Information

Checklist S1 PRISMA 2009.
(DOC)
